# Entrepreneurial Nursing interventions for the social emancipation of women in recycling

**DOI:** 10.1590/1980-220X-REEUSP-2021-0466

**Published:** 2022-02-21

**Authors:** Dirce Stein Backes, Lisiane de Borba Müller, Giovana Batistella de Mello, Mara Regina Teixeira Caino Marchiori, Andreas Büscher, Alacoque Lorenzini Erdmann

**Affiliations:** 1 Universidade Franciscana, Santa Maria, RS, Brazil.; 2 Hochschule Osnabrück, Osnabrück, Germany.; 3 Universidade Federal de Santa Catarina, Florianópolis, SC, Brazil.

**Keywords:** Pandemics, COVID-19, Community Health Nursing, Entrepreneurship, Social Responsibility, Pandemias, COVID-19, Enfermería en Salud Comunitaria, Emprendimiento, Responsabilidad Social, Pandemias, COVID-19, Enfermagem em Saúde Comunitária, Empreendedorismo, Responsabilidade Social

## Abstract

**Objective:**

To implement and signify entrepreneurial interventions in Nursing, with a view to the social emancipation of women working in an Association of Recyclable Materials.

**Method:**

Action-research with an intervention process based on an action alluding to Mother’s Day, carried out in a pandemic period, with the participation of 28 women from a Recycling Association.

**Results:**

The reflexive thematic analysis, which enabled the systematic recording of ideas, insights and the meanings of the intervention, gave rise to two categories: From apparent isolation to professional reinvention and from invisibility to dignity and the feeling of social equality.

**Conclusion:**

The interventions carried out in an Association of Recyclable Materials in a pandemic period provided, for its female workers, a sense of life, survival, dignity and empowerment, when they expected little or nothing. Enabling a social identity for the women of a Recycling Association implies, in short, overcoming linear interventions focused on assistance.

## RESUMO

**Objetivo:**

Implementar e significar intervenções empreendedoras de Enfermagem, com vistas à emancipação social de mulheres trabalhadoras de uma Associação de Materiais Recicláveis.

**Método:**

Pesquisa-ação, cujo processo de intervenção teve como cenário uma ação alusiva ao Dia das Mães, em período pandêmico, com a participação de 28 mulheres de uma Associação Recicláveis.

**Resultados:**

Da análise temática do tipo *Reflexive*, que possibilitou o registro sistemático de ideias, insights e a significação da intervenção realizada, resultaram duas categorias: Do aparente isolamento à reinvenção profissional e Da invisibilidade à dignidade e à sensação de igualdade social.

**Conclusão:**

O significado das intervenções realizadas em uma Associação de Materiais Recicláveis em período pandêmico significou, para as mulheres/Mães trabalhadoras, sentido de vida, sobrevivência, dignidade e empoderamento, quando pouco ou nada esperavam. Possibilitar uma identidade social às mulheres de uma Associação de Reciclagem implica, em suma, superar intervenções lineares e assistencialistas.

## RESUMEN

**Objetivo:**

Implementar y significar intervenciones emprendedoras de Enfermería hacia la emancipación social de mujeres trabajadoras de una Asociación de Materiales Reciclables.

**Método:**

Se trata de una Investigación-acción, cuyo proceso de intervención tuvo como escenario una acción alusiva al Día de las Madres, en período pandémico, con la participación de 28 mujeres de una Asociación de Reciclaje.

**Resultados:**

Del análisis temático de tipo reflexivo, que permitió registrar sistemáticamente las ideas, las percepciones y el sentido de la intervención realizada, resultaron dos categorías: Del aislamiento aparente a la reinvención profesional y De la invisibilidad a la dignidad y al sentido de igualdad social.

**Conclusión:**

El significado de las intervenciones llevadas a cabo en una Asociación de Materiales Reciclables durante el período pandémico les dio a las mujeres/Madres trabajadoras un sentido de supervivencia, dignidad y empoderamiento a su vida cuando esperaban poco o nada. Brindar identidad social a mujeres de una Asociación de Reciclaje implica, en definitiva, superar las intervenciones lineales y asistencialistas.

## INTRODUCTION

The Covid-19 pandemic, caused by the new coronavirus – SARS-CoV-2, triggered doubts, uncertainties, social distancing, unanswered questions and, at the same time, required professionals to reinvent themselves and led to an incessant and systematic search for strategic solutions to face multiple demands. Businesses in general were affected, but the situation got worse and magnified the already existing social problems^(1–2)^. In this context, social entrepreneurship emerged as a possible and viable alternative to support people’s needs and improve the living conditions of vulnerable populations, such as the women of an Association of Recyclable Materials, who are the object of this study.

Social entrepreneurship is complemented by the entrepreneurial movement but is distinguished from it by the prospection and implementation of innovative ideas and practical projects to achieve collective good^(3–4)^. For contemporary authors, social entrepreneurship is a new archetype of development in networks and partnerships, with a focus on human, social and sustainable dimensions. In addition, it is characterized as a mechanism of social mobilization that can support the confrontation of social problems in a creative, innovative and transforming way^(5, 6, 7)^.

Therefore, the social entrepreneur is an agent of change, that is, someone who does not only mourns for the opportunities lost due to the Covid-19 pandemic, but also envisions innovative, supportive and resilient possibilities. In the health/nursing area, social entrepreneurs are recognized for their search for innovative and sustainable solutions to practical problems and for the promotion of interactive and associative processes focused on a healthy life for individuals, families and communities^(8–9)^.

In the logic of social entrepreneurship, health must be understood as a complex system that is dynamic, self-organized and interconnected to the different social systems that aim to promote health in a social, ecological and systemic perspective^(9)^. This understanding of health is even more complex when associated with the work of the women from an Association of Recyclable Materials. Most of the time, these professionals work in unhealthy conditions, are exposed to risks of all kinds, such as chemical, biological and environmental risks, and face inequality and social devaluation^(10)^.

From this perspective, social entrepreneurship can be understood as a tool that encourages solidary and collaborative knowledge and practices, with a view to promoting comprehensive health. Researchers believe that entrepreneurship promotes processes that connect different agents and social segments, as local innovations and transformations are a result of interactive and associative networks that tackle the needs of vulnerable groups in a collaborative way^(6,11)^.

Under this approach, the work of nursing professionals cannot be reduced to linear technical-scientific skills focused only on assistance. The perception that nurses can contribute to sustainable social development through entrepreneurial care, associated with the expansion of opportunities and possibilities for individuals, families and communities, is no longer a challenge, but a prospective need for the advancement of Nursing science^(12–13)^.

This gradual and prospective entrepreneurial movement ultimately reflects the desire to overcome Cartesian approaches that are still hegemonic in health and promote a systemic thinking that can stimulate protagonism and social entrepreneurship^(14–15)^. The focus on assistance seems to have exhausted the possibilities of evolution in the face of the complex and growing social and health problems, the accelerated technological advance and the new values and ways of life, especially those resulting from the new coronavirus pandemic.

Considering the Sustainable Development Goals – 2030 Agenda, which stimulate a healthy lifestyle and well-being for all citizens, the question is: How to ensure a healthy life and contribute to the social emancipation of women who work in an Association of Recyclable Materials in the face of the Covid-19 pandemic? Therefore, the objective of this study was to implement and signify entrepreneurial interventions in Nursing, with a view to the social emancipation of women working in an Association of Recyclable Materials. 

## METHOD

### Type of Study

The action-research methodology was adopted, as it enabled the construction and intervention of knowledge and practices, with the participation of the women working in a Recycling Association and undergraduate and graduate Nursing students who participated both in the intervention process and in the investigation and understanding of the practice. The method considers the empirical knowledge based on a previously identified demand and enables interventions that can favor the social emancipation of women working in an Association of Recyclable Materials^(16)^. The Consolidated Criteria for Reporting Qualitative Research (COREQ)^(17)^ were considered in the construction of the study.

### Scenario

The intervention process was a Mother’s Day action during the pandemic period, with the participation of women from an Association of Recyclable Materials located in southern Brazil. This Association, made up mostly of female workers, has existed since 2009 and provides work and income for about 30 families that depend solely on this source of income. The number of children per family varies between four and eight. The daily work routine is eight hours, and the individual monthly income currently varies between 300 and 500 reais, with the ­sporadic addition of voluntary donations. This institution was chosen because it was the setting of an expanded action-research ­focused on the social entrepreneurship of Nursing professionals, conducted by the main author, and because, in the pandemic context, the local leadership wanted to provide a surprise Mother’s Day event for the workers of this Association. 

### Population

The 28 women who work at the Association of Recyclable Materials participated in the intervention process, and 16 women, four undergraduate students and two graduate Nursing students participated in the process of signifying the interventions. The following inclusion criteria were considered: women with more than two years of experience in the Association and undergraduate and graduate Nursing students who had previously participated in the interventions in the Association. Women and students who did not attend the interviews on the days and times previously scheduled were excluded from the study.

### Interventions in the Association of Recyclable Materials

At the beginning of the action-research, from February to July 2021, interventions were carried out in the workplace of the 28 women. The interventions based on a previous survey and carried out in the face-to-face modality included: weekly educational workshops on health promotion; activities ­related to risk prevention at work; basic care to combat the new coronavirus; among others. 

This study, however, will address and discuss only the ­meanings of the interventions related to the Mother’s Day tribute, held in May 2021, during the Covid-19 pandemic. The surprise Mother’s Day tribute, promoted by researchers and undergraduate and graduate Nursing students, was carried out with the consent of the local leadership, in a reinvented face-to-face modality, respecting the sanitary protocols. The tribute lasted two hours and consisted of virtual messages presented live, in an outdoor space, with repercussions on the local community, and in the delivery of roses, a personal letter, a special lunch box and a personalized gift (Covid-19 protection kit) to each of the women/Mothers. The personal letter contained the main qualities and abilities of each woman/Mother. 

### Data Collection

After systematizing the activities in the study setting, the investigative process of signifying the intervention was conducted through individual interviews with the 16 women and the six undergraduate and graduate Nursing students previously selected and contacted. The interviews were carried out on days and times previously scheduled with the participants and were based on a guiding question that was discussed in depth: Tell me about the meaning of the Mother’s Day tribute. What did this moment mean to you?

### Data Analysis

Reflexive Thematic Analysis was adopted, which enabled the systematic recording of ideas and insights and a fluid and flexible codification of the meanings investigated. This process aimed not only to achieve accuracy, but also to deepen the immersion in the data. For this purpose, the six phases of the Thematic Analysis were followed: Familiarization through repeated readings of the data and a drafted list of ideas; Generation of initial codes, manually, by systematizing relevant extracts; Search for themes based on the classification of different codes; Refinement of the themes with the validation of the initial themes; Naming the themes based on the essence of each of them in their set of codes; and Elaboration of the report that offered a reflective description of the experience^(18)^.

### Ethical Aspects

The recommendations of Resolution No. 466/2012 of the National Health Council^(19)^ and of Curricular Letter No. 2 of 2021^(20)^ on research in times of pandemic were followed. The study was approved by the Research Ethics Committee # 4.253.910/2020. After the acceptance of the participants, they signed an Informed Consent Form. To maintain anonymity, the speeches of the participants were identified in the text with the letters “M” for Mother and “S” for student, followed by a number, corresponding to the order of the interviews: M1, M2... M16 ; US1, US2...S4; GS1 and GS2.

## RESULTS

The organized and analyzed data resulted in two thematic categories: From apparent isolation to professional reinvention and from invisibility to dignity and the feeling of social equality. However, beyond the linear narration, the description of the themes aims to give voice to feelings, experiences and expressions manifested and/or not by the participants, considering the interactive process of the action-research.

### From Apparent Isolation to Professional Reinvention

While several services were closed and/or operated partially under sanitary protocols, recycling workers were not so lucky. To maintain the livelihood of their families, they had to keep working in even more unhealthy and exhausting conditions, as they could not know, for sure, the form of proliferation of the Covid-19 virus, the origin and conditions of the material received, among other problems, uncertainties and prerogatives. What to expect on Mother’s Day, when issues kept them on edge and uncertainties paralyzed expectations, goals and dreams?

In the midst of all these questions and uncertainties, the researchers, in dialogue with the local leadership, asked themselves: how to celebrate Mother’s Day with women working in the Association of Recyclable Materials? What to provide to these mothers, who are usually so invisible to society and even more invisible in the face of strict social distancing protocols? Until then, the only certainty was that the Mother’s Day would not go unnoticed. But how to reinvent it, considering that in previous years this day had always been celebrated in an expressive way and at the university? How to get the collaboration of traditional partners, such as makeup institutes, steakhouses and transport companies?

In this context of reflections and understandings, an idea emerged: presenting a live virtual message for the members of the association, in an outdoor space, on a surprise day and time. This moment, however, was not limited to the message, which alone already had a special meaning, but also mobilized the neighborhood and the local community. In just a few minutes, several mothers from the community had joined the women of the Association to reinforce the importance of their work to the community. The feeling of each of the mothers was stamped in their face, in their expression, in the gestures and attitudes, as if they were saying “*I never thought that I would be remembered this year*”. The outdoor virtual message with local repercussions made them even more special and distinct in the community and the celebration was even bigger than the previous editions, done before the pandemic, as they expressed “*this was the most beautiful tribute we have ever received*”.

A striking sentence with a remarkable meaning was: “*I never thought I would be remembered on this Mother’s Day*”. This idea was expressed by almost all the women/Mothers of the Association, which, in this context, transcends words and linear understandings. The fact that they were remembered as Mothers during the Covid-19 pandemic empowered them as women and workers and provided them with a status of personal, professional and social dignity.

*I never thought I would be remembered on this Mother’s Day*. (M1, M2, M3, M5, M6, M9, M11, M13)

This day was remembered as “*special, we are not alone*” by the Mothers and also by the undergraduate and graduate Nursing students who shared this reinvention, and who, even without hugs, experienced the love, affection and tenderness and felt welcomed and comforted in a period of social distancing. For two students, this reinvention and the feeling of solidarity will be forever ingrained in their memory, as follows:

*Every day that passes I am more convinced that I am on the right path. Being with someone who prioritizes people’s humility. This is what I want in my nursing profession. I have no words to express what I feel, especially after everything I’ve heard from these mothers. This feeling made me stronger.* (US2)

*I can’t find the words to describe the feelings that went through my heart. I just want to thank you for sharing these moments, and making a difference in the lives of these mothers. I learned a lot and I feel I can contribute a lot more.* (GS1)

Both the Mothers and the members of the project perceived and experienced the synergy of the moment, which was not only a tribute; it showed presence, reception, empathy, solidarity; it was the expression of Nursing care in its true entrepreneurial spirit. In the solidary and collaborative exchange of knowledge and practices, everyone was invigorated, everyone grew and learned, which made social isolation more tolerable and less painful. 

### From Invisibility to Dignity and the Feeling of Social Equality

The speeches of the Mothers demonstrated that social entrepreneurship in Nursing is not reduced to “*doing things*” or simply promoting innovations. Beyond “*doing things*” or ­promoting changes, people want to be heard, welcomed and respected in their uniqueness and dignity. It was noted that it was necessary to quickly deconstruct knowledge and assistance practices to reach the true meaning of “*professional reinvention*”, so that the interventions were meaningful for the Mothers and also for the students involved.

A statement that gave rise to feelings, expressions and long discussions was “*this was the first time that I got roses on Mother’s Day*”. This was expressed by one of the Mothers and reiterated by others several times, so the idea recurrently returned to the theoretical-practical reflections of the project members. For these Mothers, getting roses was not reduced to receiving a gift or donation, but felt like the recognition of existence, dignity, respect and, above all, equality with Mothers from other social classes, for whom getting roses represents a normal/natural gesture. For the Mothers of the association, the roses represented recognition and confirmation of their identity, their name, and their equal value in society.

The force of the expression “*this was the first time that I got roses on Mother’s Day*” also shows the process of social exclusion reflected in the Mother’s feelings. While most women in society get roses from their children on Mother’s Day, the women/Mothers of the association are often deprived of this simple human gesture, due to lack of financial conditions or the impossibility of being close to their children. The feeling of rescuing the identity and dignity of being a mother is expressed in the statement:

*We didn’t even expect anything or we thought that it would be ­something simple, just a little gift due to the pandemic. But in the end it was all beautiful, heartwarming. I felt like a mother. It’s a good thing that we can receive roses, receive affection. When I saw other mothers getting roses on television, I always asked myself: ‘why them and not me’? But today I realized that I am also special. We know that all these things were chosen with love.* (M14)

In the speeches of other Mothers, the meaning of this ­manifestation of recognition and empathy in times of social ­isolation was evident. The main feelings were of not being alone, of having someone to share their pain, anxieties, fears, ­uncertainties and achievements, as expressed in the ­following report:

*We need to work so we have something to eat. We have no choice. We are scared, we are, but we are strong, we fight, we take care of each other and we encourage each other. Today’s tribute filled us with energy, vivacity, strength and courage. We feel that we are not alone and forgotten. We have people who love us, value us and are interested in us. Thank you for the beautiful tribute, the affection and the gifts.* (M5)

These social and scientific interactions do not only ­encompass those who receive the tribute, but uplift all those involved in the process of (re)creation and promotion, as in the case of undergraduate and graduate students. In this process, there was a real exchange of energy, joy, emotions and theoretical and practical learning, as expressed in the following two statements:

*These moments uplift our heart. The importance of these women’s work makes us reflect that, with small gestures and attitudes, it is possible to transform our community into a better place. Their love transcends the challenges of social distancing and warms our hearts*. (US4)

*I am grateful for having participated in the Mother’s Day ­tribute for these amazing and hardworking women. I am grateful for ­having known each of them, for learning with them every day, for having the opportunity to share knowledge and to see them so happy and excited. It was extremely important for me, as an academic, to carry out this action, to feel the joy and emotion of each mother*. (GS2)

The feeling of social equality, as expressed by the Mothers of the Association, is not only related to professional status, a compatible salary or a renowned position. For the Mothers of this Association, being seen, remembered and honored when they expected nothing or little, provided a sense of life, survival, sustainability and eternity. At the same time, for those involved in the intervention, being able to provide a solidary and singular moment to the Mothers provided a sense of distinction and the feeling of contributing to a more just and egalitarian society. 

## DISCUSSION

When discussing social entrepreneurship in Nursing in a pandemic period, the first questions that come to mind are: How to think about social entrepreneurship in the face of the serious crisis in global health? How to think about social entrepreneurship in the face of estimates that indicate that the number of poor people jumped from 9.5 million to more than 27 million in February 2021, giving rise to scenarios of instability in people’s lives? What new skills and professional competences need to be developed and implemented by Nursing? How to develop an entrepreneurial culture in Nursing/Health, in order to overcome healthcare models focused on assistance and contribute to healthy and sustainable development?

Every crisis can create opportunities and induce innovation and reinvention A scenario of uncertainty and chaos, such as the pandemic of the new coronavirus, can strengthen and expand people’s curiosity and their ability to form prospective connections in search of agile and collective solutions. Therefore, Nurses must be able and willing to explore opportunities, knowing that regrets, disorders and paralysis may appear. During the pandemic, more than in other times, people got out of their comfort zone and professional reinventions were made possible. In this context, it is necessary to gradually promote collective and interprofessional processes that can expand partnerships and strengthen skills such as resilience, patience, tolerance, innovation and the ability to listen with empathy^(14)^.

We are, therefore, living a pandemic made simultaneously of order, disorder and (re)organization, in addition to alternations between certainties and uncertainties^(15)^. Therefore, in nursing, as in all sectors of society, reinventions were incessant and prospective, perhaps as never before. Nursing has reinvented itself in the contexts of teaching, research, hospitals, clinics, basic health units, homes, that is, in the most different spaces and areas of professional activity^(21)^. But how has this reinvention occurred in community activities? What are the breakthroughs and advances in social entrepreneurship in Nursing? 

When they mention “*today’s tribute filled us with energy, vivacity, strength and courage. We feel that we are not alone and forgotten. We have people who love us, value us and are interested in us*”, which professionals and what care are they talking about? We immediately think that Nursing care transcends geographic, social, cultural and linguistic spaces and barriers. Has the Nursing Now campaign managed to demonstrate and promote the true meaning of Nursing care in its community and social context? What can and should be different according to social entrepreneurship?

In this context, an entrepreneurial Nursing care is ­materialized in an attentive and sensible look, in the ability to perceive and do the difference, in the power to reinvent rhetoric, discourses and processes. Finally, it is in the attitude of ­leaving the commonplace and taking the risk of, eventually, being exposed to “crowds” in the face of strict social isolation protocols. Under this (re)created approach, Nursing care can only be understood in the light of complex thought, that is, as a complex phenomenon, systematized by multiple relationships, interactions and systemic associations, aimed at promoting a comprehensive care integrated with its surroundings^(9,14)^.

The expression “*this was the first time that I got roses on Mother’s Day*” shows, on the one hand, vertical actions and ­attitudes that boil down to giving out what can be spared and, on the other hand, the expectations, usually frustrated, of underprivileged people, used for utilitarian purposes. Based on these evidence, social entrepreneurship demonstrates that it is necessary to go beyond assistance interventions focused on giving, on intervening at any cost or on editing norms and guidelines for others. Corroborating this idea, studies show that social entrepreneurship is related to the coexistence of humans, with the purpose of enabling a social identity through a ­permanent process of metamorphosis and providing a sense of existence and history through the realization of a future with and among other human beings^(22, 23, 24)^.

In addition to strengthening prospective and entrepreneurial leadership, Nursing must also be able to expand its social influence and disseminate its knowledge and community practices^(11–12)^. In this context, the contributions of this study to the technical and scientific advancement of Nursing are related to the perception that Nurses are dynamic, agile and flexible professionals that can reinvent themselves. In short, the experience of the outbursts during the Covid-19 pandemic demonstrates that Nursing professionals, as well as other health professionals, need to be able and willing to find opportunities under new perspectives and to contribute in proactive and prospective manners to achieve the Sustainable Development Goals^(25)^ at a local and global level.

A limitation of this study was the restriction of time and space, and the limited participation of students and ­people close to the women/Mothers of the association on the Mother’s Day intervention. Another limitation was the constant surveillance of sanitary protocols to avoid contamination by the new coronavirus. 

## CONCLUSION

The interventions carried out in an Association of Recyclable Materials in a pandemic period provided, for its female workers, a sense of life, survival, dignity and empowerment when they expected little or nothing. And, for undergraduate and graduate Nursing students, the interventions represented the possibility of creative, daring and transformative reinvention. 

It was shown that the feeling of social equality is not solely related to professional status, a compatible salary or a ­renowned position. For the Mothers of the Association, being seen, remembered and honored represented an uplifting and emancipating force. At the same time, for undergraduate and graduate Nursing students, being able to provide a solidary and singular moment to the Mothers created a feeling of distinction and contribution to a more just and egalitarian society.

Following the results of this study, new theoretical and practical investigations on the theme should be carried out to expand and strengthen the entrepreneurial culture of nurses in the community and social context. It is essential that, beyond the pandemic period, professional reinvention and the ability to envision unprecedented possibilities prevail in places where most people only see problems.
